# Neurodegeneration and Vision Loss after Mild Blunt Trauma in the C57Bl/6 and DBA/2J Mouse

**DOI:** 10.1371/journal.pone.0131921

**Published:** 2015-07-06

**Authors:** Courtney Bricker-Anthony, Tonia S. Rex

**Affiliations:** 1 Vanderbilt Eye Institute, Vanderbilt University, Nashville, Tennessee, United States of America; 2 Vanderbilt Brain Institute, Vanderbilt University, Nashville, Tennessee, United States of America; University of Florida, UNITED STATES

## Abstract

Damage to the eye from blast exposure can occur as a result of the overpressure air-wave (primary injury), flying debris (secondary injury), blunt force trauma (tertiary injury), and/or chemical/thermal burns (quaternary injury). In this study, we investigated damage in the contralateral eye after a blast directed at the ipsilateral eye in the C57Bl/6J and DBA/2J mouse. Assessments of ocular health (gross pathology, electroretinogram recordings, optokinetic tracking, optical coherence tomography and histology) were performed at 3, 7, 14 and 28 days post-trauma. Olfactory epithelium and optic nerves were also examined. Anterior pathologies were more common in the DBA/2J than in the C57Bl/6 and could be prevented with non-medicated viscous eye drops. Visual acuity decreased over time in both strains, but was more rapid and severe in the DBA/2J. Retinal cell death was present in approximately 10% of the retina at 7 and 28 days post-blast in both strains. Approximately 60% of the cell death occurred in photoreceptors. Increased oxidative stress and microglial reactivity was detected in both strains, beginning at 3 days post-injury. However, there was no sign of injury to the olfactory epithelium or optic nerve in either strain. Although our model directs an overpressure air-wave at the left eye in a restrained and otherwise protected mouse, retinal damage was detected in the contralateral eye. The lack of damage to the olfactory epithelium and optic nerve, as well as the different timing of cell death as compared to the blast-exposed eye, suggests that the injuries were due to physical contact between the contralateral eye and the housing chamber of the blast device and not propagation of the blast wave through the head. Thus we describe a model of mild blunt eye trauma.

## Introduction

Blast exposure can cause damage to the eye as a result of the primary blast wave, perforating or penetrating injuries from shrapnel and debris (secondary), blunt force trauma from being thrown against a hard surface (tertiary), and/or thermal and chemical burns (quaternary) [1]. The eye, an exposed organ, is particularly vulnerable to injury. As Gendler et al., nicely articulated, “The eye represents 0.1% of the total body surface yet it accounts for 8–13% of battle injuries in modern warfare [2]”. Ocular injuries are the fourth most common injury in the recent wars in Iraq and Afghanistan [3]. In one recent study of veterans with traumatic brain injury, 66% were diagnosed with ocular trauma, mostly due to blast exposure [4]. In addition, in the civilian population, 50,000 U.S. citizens lose vision every year as a result of blunt force trauma [5]. Despite varying injury mechanisms, both blast and blunt ocular trauma patients share similar pathology, including corneal abrasions, cataracts, intraocular foreign bodies, retinal detachments, vitreous hemorrhage, retinal pigment epithelium (RPE) disruption and optic neuropathy [3–9].

In order to determine if the blast wave alone affects the eye directly, we developed a model that directs an overpressure air wave to the left eye while the head, body, and contralateral eye are protected [7]. We previously characterized the injury response in the blast-exposed eye in C57Bl/6 (Bl/6) and DBA/2J (D2) mice [8,9]. In the blast-exposed Bl/6 eye, cell death, alterations in the electroretinogram (ERG) and decreased spatial acuity thresholds were detected at 1 month post-blast [8]. In the blast-exposed D2 mouse eye, cell death was first detected at 3 days post-injury and decreases in the ERG were first detected at 7 days post-blast [9]. A large neuroinflammatory response including recruitment of inflammatory cells occurred in the in the D2, but not Bl/6 blast-exposed eyes, likely due to the lack a regulatory mechanism of immune privilege in the anterior chamber (anterior chamber-associated immune deviation) of the D2 mice [10]. Acute treatment with non-medicated eye drops protected the cornea and limited the retinal injury response, but had no effect on optic nerve degeneration or vision loss, demonstrating that these deficits were a direct result of blast exposure.

In our model, the head, contralateral eye and body are shielded from the over-pressure air-wave. Yet we detect vision loss in the contralateral eye. The purpose of this study was to determine if the vision loss in the contralateral eye (herein referred to as “eye”) was due to blunt trauma or propagation of the air-wave through the tissue and we characterized the cellular and molecular changes in the eyes of both mouse strains during the first month post-blast. We chose to explore this complex injury in two mouse strains in order to gain insight into the potential mechanisms underlying vision loss, and because there is significant variability between strains suggesting that no single strain is ideal for modeling human trauma. This is exemplified in the myriad of ongoing studies using recombinant inbred mice showing wide phenotypic variability that can be mapped to specific genetic loci [11–13]. The Bl/6 was chosen because it is the most commonly used laboratory mouse strain and is the background for many transgenic lines. The D2 was chosen because it lacks a molecular component necessary for a fully functional ocular immune privilege, and contains reactive microglia even at the pre-glaucomatous ages used in this study. Thus, if neuroinflammation plays an important role in the retinal response to trauma we might expect more vision loss in the D2 than the Bl/6 after trauma.

## Materials and Methods

### Animals

Two strains of commercially available mice, Bl/6 (n = 44) and D2 (n = 49), were used in this study (The Jackson Laboratory, Bar Harbor, ME). All mice were between 8 to 12 weeks of age. Mice were housed together (maximum 5 mice per cage) in a non-barrier facility and were maintained on a 12hr light/dark cycle and provided food and water *ad libitum*. When mice arrived in the facility, each cage was assigned to a specific time point for collection (3, 7, 28 dpi or naïve control) and mice within the cages were assigned numbers. The majority of D2 mice develop an elevated intraocular pressure at 6–9 months of age followed by axon degeneration and retinal ganglion cell (RGC) death [14]. To avoid complications in our study, we only used D2 mice at 2–4 months of age, prior to development of glaucoma. All experimental procedures were approved by the Institutional Animal Care and Use Committee of Vanderbilt University and were in accordance with the Association for Research in Vision and Ophthalmology Statement for the Use of Animals in Vision and Ophthalmic research.

### Ocular Blast Injury

Blast was performed as previously described [7]. Briefly, Bl/6 (n = 38) and D2 (n = 42) mice were anesthetized with an intraperitoneal injection of ketamine/xylazine (105/8 mg/kg on average), secured and padded within a housing chamber, and the left (ipsilateral) eye of the mouse was positioned against the hole in the pipe, which was aligned with the barrel of the paintball marker. We performed all experiments at a blast pressure of 26psi in the morning. For the first 3 days post-injury (dpi), mice were provided gel recovery food (Clear H_2_O, Portland, Maine, United States). The Bl/6 sample sizes for each time point are as follows: 3 dpi (n = 7), 7 dpi (n = 8), 28 dpi (n = 13) and naïve controls for immunohistochemistry and histology (n = 8). The D2 sample sizes for each time point are as follows: 3 dpi (n = 11), 7 dpi (n = 3), 28 dpi (n = 13) and naïve controls for immunohistochemistry and histology (n = 7). An additional 10 Bl/6 and 15 D2 mice exposed to a 26psi blast were used to bolster our sample sizes for assessments of visual function, but they were collected at later time points for other studies. Only contralateral eyes were used for this study and are simply referred to as “eyes” for the remainder of the paper.

### Gross Pathology

The eyes of awake Bl/6 (n = 38) and D2 (n = 42) mice were assessed prior to and 0, 3, 7, 14 and 28 dpi using an SZX16 stereomicroscope (Olympus, Center Valley, Pennsylvania, United States). Representative images were taken using a DP71 camera (Olympus, Center Valley, Pennsylvania, United States). Eyes were examined for the presence of corneal abrasions, corneal edema, calcium deposits, hyphemas, cataracts and corneal neovascularization.

### Ultra-high Resolution Optical Coherence Tomography (OCT)

Bl/6 (n = 13) and D2 (n = 10) mice were anesthetized with an intraperitoneal injection of ketamine/xylazine (25/10 mg/kg). A 1% tropicamide solution was used to dilate the eyes for retinal imaging; no dilation drops were used for anterior chamber imaging. Genteal lubricating eye gel (Alcon, Fort Worth, Texas, United States) was used to keep the eyes moist. The mice were wrapped in gauze, placed in a holding chamber, and head position was stabilized with a bite bar. A Bioptigen ultra-high resolution spectral domain OCT system with a mouse retinal bore (Bioptigen LLC, Morrisville, North Carolina, United States) was used to image the retinas. A 12mm telecentric bore was used to image the anterior chamber in Bl/6 mice (n = 7). Anterior chamber depth (from the inner portion of the cornea to the top of the lens) was measured using calibrated digital calipers in InVivoVue software (Bioptigen LLC, Morrisville, North Carolina, United States).

### Visual Acuity

The Optomotry optokinetic nystagmus (OKN) system (Cerebral Mechanics, Canada) was used to assess photopic visual acuity in awake Bl/6 (n = 16) and D2 (n = 9) mice at baseline, 3, 7, 14, and 28 dpi in the afternoon. The testing paradigm was step-wise and the experimenter was masked to the testing conditions. Mice were acclimated to the testing chamber for 5 minutes prior to the initiation of each test. Spatial frequency for visual acuity was 0.042 c/d.

### Electroretinogram (ERG)

A Diagnosys LLC Espion Electrophysiology system with heated mouse platform (Lowell, MA) was used to perform flash ERGs baseline, 7 and 28 dpi in the afternoon. Dark-adapted Bl/6 (n = 22) and D2 (n = 26) mice were anesthetized with ketamine/xylazine (25/8 mg/kg) and eyes were dilated with a 1% tropicamide solution. In a dark room with only a dim red light source, mice were exposed to flashes of light ranging from -2 to 2.88 log cd*s/m^2^ with a flash frequency of 2000Hz. For flashes below -1 log cd*s/m^2^, the inter sweep delay was 10 sec, for the -1 log cd*s/m^2^ flash it was 15sec, and for all remaining flashes, the delay was 20 sec. Oscillatory potentials were measured at 3 log cd*s/m^2^ sampled at 2000Hz with an inter sweep delay of 15sec. Amplitudes were measured from baseline to peak. The b_max_ to a_max_ ratio was calculated for each mouse at the 1 log cd*s/m^2^ flash intensity.

### Tissue Collection

Mice were euthanized by a 1ml overdose of Avertin (Sigma-Aldrich, St. Louis, Missouri, United States) delivered via intraperitoneal injection and transcardially perfused with 4% paraformaldehyde (PFA, Electron Microscopy Sciences, Hatfield, Pennsylvania, United States) and phosphate buffered saline (PBS). The tissues were collected and incubated in either 4% PFA (for immunohistochemistry, eyes and olfactory epithelium; Bl/6 n = 32, D2 n = 33) or 4% PFA with 0.5% glutaraldehyde (Electron Microscopy Sciences, Hatfield, Pennsylvania, United States; for resin, eyes and optic nerves; Bl/6 n = 7, D2 n = 9).

### Retina Histology

For histological analysis, eyecups (Bl6 n = 7; D2 n = 9) were bisected to allow infiltration of Spurr’s resin (Electron Microscopy Sciences, Hatfield, Pennsylvania, United States), sectioned at 1 micron-thickness on a Reichardt-Jung Ultracut E microtome (Leica Microsystems, Vienna, Austria) and stained with toluidine blue. Representative images were collected on an Olympus Provis AX70 (Olympus, Center Valley, Pennsylvania, United States) with a 60x oil objective lens. To quantify RPE damage, a grading scale was developed to classify the vacuoles: 1 (normal, very infrequent and small); 2 (small and infrequent); 3 (small and frequent); 4 (large and infrequent); and 5 (large and frequent). The number of pyknotic nuclei in the outer nuclear layer (ONL) or inner nuclear layer (INL) was quantified within a single section of retina through the middle of each eye. The tissue was graded and assessed by a blinded experimenter.

### Optic Nerve Histology

Bl/6 (n = 5) and D2 (n = 7) optic nerves were placed in 1% osmium tetroxide in 0.1 M cacodylate buffer, dehydrated in a graded ethanol series and embedded in Spurr’s resin (Electron Microscopy Sciences, Hatfield, PA). Starting from the proximal (near the eye) end of the optic nerve, 1 μm-thick sections were collected (Reichert-Jung Ultracute E; Leica Microsystems, Vienna, Austria) and stained with 1% p-phenylenediamine in 50% methanol (Sigma-Aldrich, St. Louis, Missouri, United States). Sections were imaged on an Olympus Provis AX70 microscope using a 100x oil immersion objective lens. Imaging was performed in a masked fashion.

### Retina and Olfactory Epithelium Immunohistochemistry

For immunohistochemistry, Bl/6 (n = 32) and D2 (n = 33) eyes and olfactory epithelium (Bl/6 n = 4) were cryo-protected in 30% sucrose overnight at 4°C, embedded in Tissue Freezing Medium (Triangle Biomedical, Durham, NC) and then sectioned on a cryostat (Fisher, Pittsburgh, PA). We collected 10 μm-thick sections on 12 slides, with each slide containing representative sections from the entire eye. The sections were rinsed with PBS and incubated at room temperature in normal donkey serum at 1:20 in 0.1 M phosphate buffer with 0.5% bovine serum albumin and 0.1% Triton X 100 (PBT) for 2 hours, followed by an overnight incubation at 4°C in primary antibody in PBT (Table 1). The sections were rinsed with PBS and incubated with a secondary antibody at a 1:200 dilution (Life Technologies, Grand Island, NY) for 2 hours at room temperature, rinsed with PBS and mounted in Vectashield Mounting medium with DAPI (Vector Laboratories, Burlingame, CA). Imaging was performed on a Nikon Eclipse epifluorescence microscope (Nikon, Melville, NY) or an Olympus FV-1000 confocal microscope (Olympus, Center Valley, PA). Imaging on the Olympus FV-1000 microscope was performed through use of the VUMC Cell Imaging Shared Resource. The tissue was assessed in a masked fashion.

**Table 1:** Primary antibodies used.

Antigen	Type	Host	Dilution	Manufacturer	Catalog number
Caspase-1	Polyclonal	Rabbit	1:100	Millipore, Billerica, MA	AB1871
GFAP	Polyclonal	Rabbit	1:400	Dako, Carpenteria, CA	Z0334
Iba1	Polyclonal	Rabbit	1:500	Wako, Richmond, VA	019–19741
Nitrotyrosine	Polyclonal	Rabbit	1:500	Millipore, Billerica, MA	06–284
RIP1	Polyclonal	Rabbit	1:100	Santa Cruz, Santa Cruz, CA	sc-7881
RIP3	Polyclonal	Goat	1:100	Santa Cruz, Santa Cruz, CA	sc-47364

### TUNEL Quantification

Retina (Bl/6 n = 20; D2 n = 16) and olfactory epithelium (Bl/6 n = 4) sections were labeled with the TUNEL Apoptosis Detection Kit adhering to the manufacturer’s protocol (Merck Millipore, Darmstadt, Germany) and mounted with Vectashield Mounting Medium with DAPI. TUNEL positive cells within the ONL, INL and GCL were counted and the lengths of the regions with TUNEL positive cells (affected regions) were measured using NIS Elements Advanced Research software (Nikon, Melville, NY). The total length of each retinal section with TUNEL positive cells (affected section) was also measured. In order to determine the percentage of the retina with cell death, we measured and summed the lengths of all sections on the slide. Then, we divided the sum of affected region lengths by the total length of all sections and multiplied this value by 100, which yielded the percentage of retina with TUNEL positive cells. Quantification was performed in a masked fashion.

### Statistical Analysis

The average ± SEM were calculated and presented for each data set. A one-way ANOVA with a Bonferroni’s post-hoc test was performed on the visual acuity and ERG data using GraphPad Prism software (GraphPad, La Jolla, CA).

## Results

### Anterior segment injuries present in the D2, not Bl/6 eye

**Fig 1:**
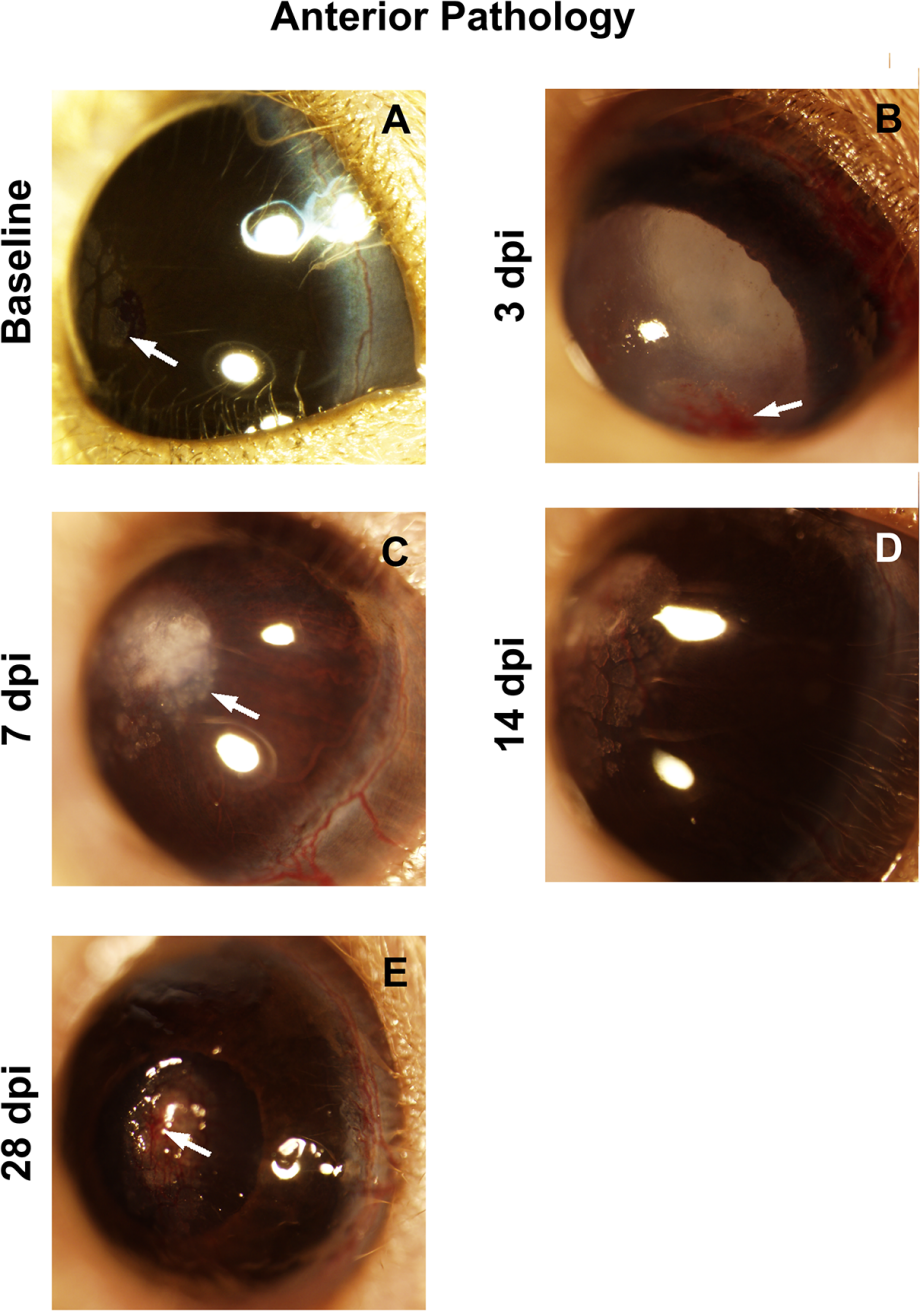
The ocular surface of the D2 eye is injured. (A) Calcium deposits (arrow) are common in the normal D2 eye. (B) Hyphema (arrow), corneal edema and a cataract at 3 dpi. (C) Corneal edema and calcium deposits (arrow) at 7 dpi. (D) Corneal edema, corneal neovascularization and calcium deposits at 14 dpi. (E) A corneal scar and corneal neovascularization (arrow) at 28 dpi.

No anterior pathology was detected in the Bl/6 eye, consistent with results in the blast-exposed eye and naïve controls.[8] In the D2 eye, corneal edema occurred in 52% of eyes at 3 days dpi (n = 27) and remained in 43% of mice at 28 dpi (n = 7, Fig 1B). Cataracts were observed in 30% of D2 eyes at both 3 and 28 dpi (Fig 1B). Pathologies that became more prevalent later included corneal neovascularization and corneal calcifications. Corneal neovascularization was detected in 44% and 29% of eyes at 14 (n = 9) and 28 dpi, respectively (Fig 1D and 1E). Corneal calcification was detected in 33% and 43% of eyes at 14 and 28 dpi, respectively (Fig 1E). Note that calcium deposits in the cornea were present in many of the D2 blast and naïve control mice as a normal finding for this strain (Fig 1C and 1D) [15]. Similar to our previous results in the blast-exposed eye, treatment with non-medicated eye drops prevented all of the corneal pathology. Thus, we treated all following mice with non-medicated eye drops to prevent corneal pathology, unless otherwise stated in order to test visual function.

### Ocular trauma causes retinal detachments

**Fig 2:**
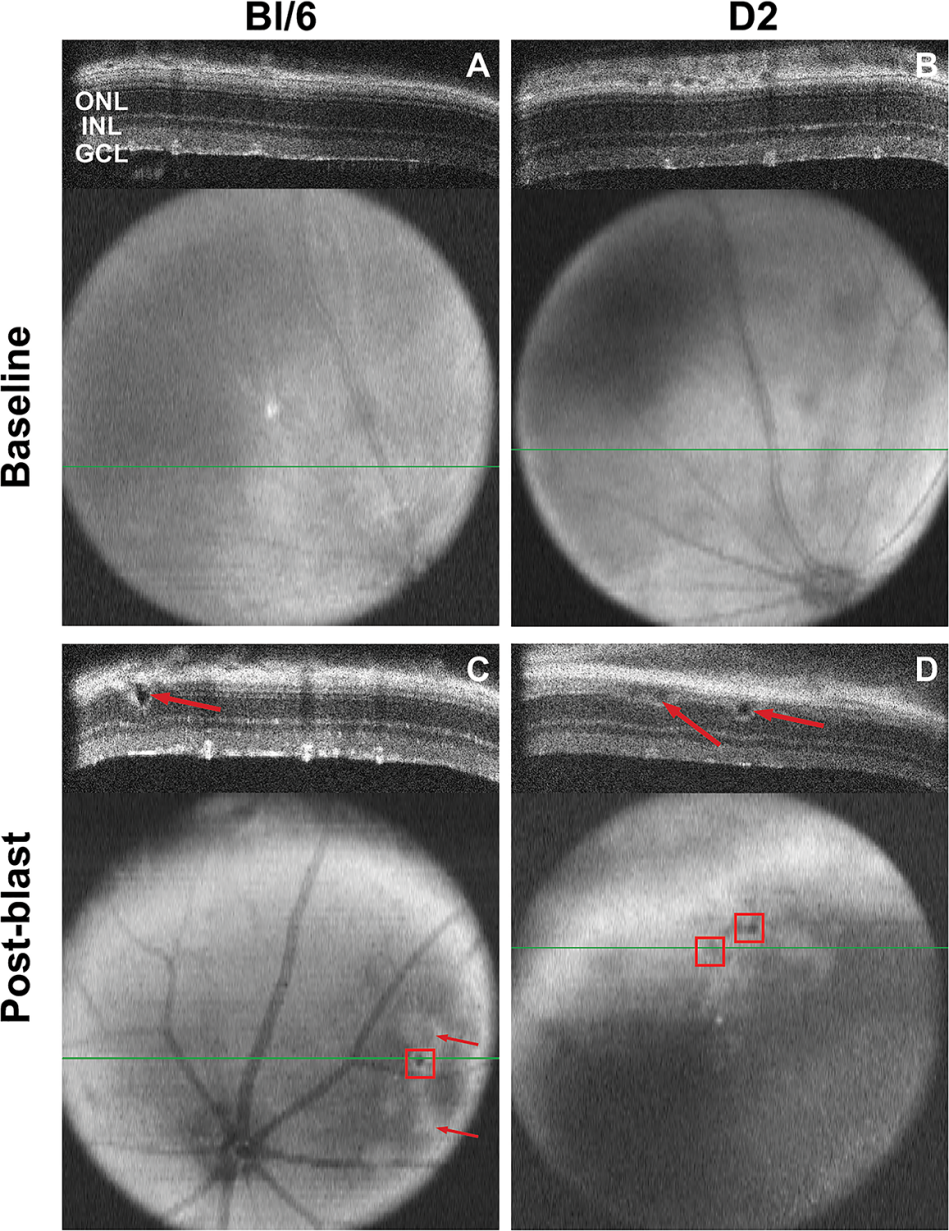
Retinal detachments occur in both Bl/6 and D2 retinas. The Bl/6 (A) and D2 (B) retinas appear normal at baseline in both the b-scan and fundus images. The green line in the fundus image denotes the location of the b-scan. (C) A retinal detachment (arrow) is present at 7 dpi in the Bl/6 retina and is visible on the fundus image as a small, round dark area (red box). Areas of outer segment disruption appear as white, patchy areas on the fundus image (C, arrows). (D) Retinal detachments in a D2 retina at 14 dpi (arrows, red boxes).

In the Bl/6 mouse, disruption of the outer segments (as determined by a bright area on the fundus, (Fig 2C) was observed in 25% of retinae within the mid-periphery at 7 days (n = 8) and in 8% of retinae at 14 (n = 13) and 28 dpi (n = 11). Retinal detachments were detected in 13% and 15% of Bl/6 retinae at 7 and 14 dpi, respectively (Fig 2C). This decreased to 8% of retinas at 28 dpi. Up to 8 retinal detachments per eye were detected and the average height of the retinal detachments was 0.02±0.003mm at all time points.

In the D2 mice, no retinal detachments were detected at 7dpi (n = 10). At 14 dpi (n = 5), an average of 13 retinal detachments per eye were detected in 17% of eyes (Fig 2D). The average height of the retinal detachments was 0.04±0.07mm. No retinal detachments were detected at 28 dpi (n = 8) suggesting that they resolved over time.

### Trauma damages both the neural retina and RPE

**Fig 3:**
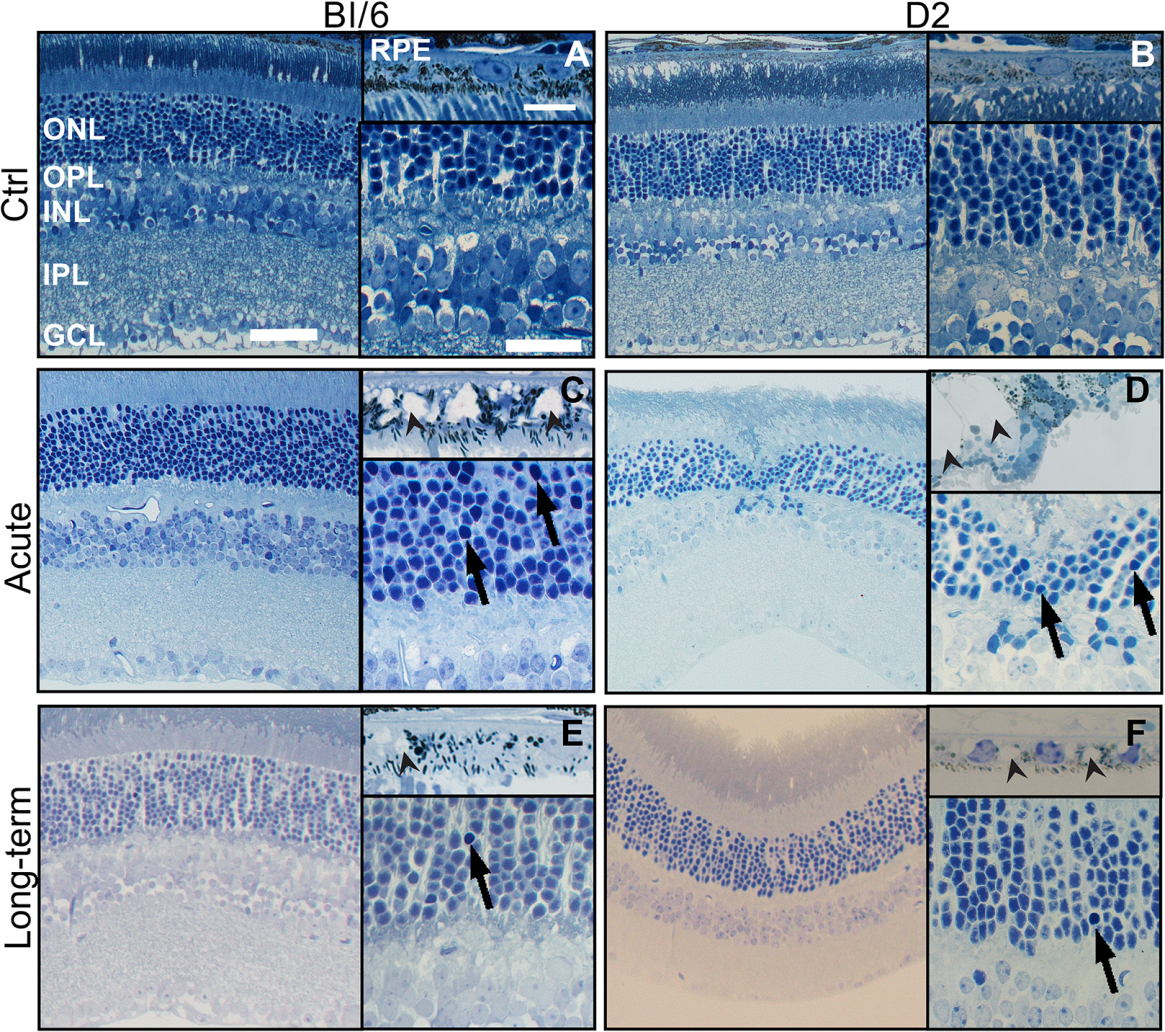
Focal retinal and RPE damage occurs in the eyes of both strains post-blast. Neither Bl/6 (A) nor D2 (B) control RPE and retina contains vacuoles or pyknotic nuclei. (C) At 7 dpi, there are grade 3 vacuoles in RPE (arrowheads), as well as small clusters of pyknotic nuclei in the mid-peripheral retina of the Bl/6 eye (arrows). (D) At 3dpi in the D2, grade 5 vacuoles are present in the RPE (arrowheads) and pyknotic nuclei are present in areas of retinal detachment (arrows). (E) At 28 dpi in the Bl/6, RPE vacuoles (arrowhead) are reduced in size to grade 1 and pyknotic nuclei (arrow) are rare. (F) The D2 eye contains grade 2 RPE vacuoles and no pyknotic nuclei at 28 dpi. The scale bars are 5μm in the RPE images, 50μm in the low magnification retina images and 25μm in the high magnification retina images.

Naïve controls for both strains had healthy RPE with no vacuoles and retinas that lacked pyknotic nuclei or other pathology (Fig 3A and 3B). After blast, while much of the retina looked normal, focal damage was detected in eyes from both strains.

Pyknotic nuclei were present in Bl/6 eyes post-blast (Fig 3C and 3E). The average number of pyknotic nuclei at 7 dpi (n = 3) was 7±2 cells/mm retina in the ONL and 10±3 cells/mm retina in the INL. The number decreased to 1±1 cells/mm in both the ONL and INL at 28 dpi (n = 3). Grade 3 vacuoles were observed in all eyes at 7 dpi (Fig 3C). At 28 dpi, the RPE was normal (grade 1) in all of the eyes examined (Fig 3E).

In the eyes of D2 mice, pyknotic nuclei were also observed post-injury (Fig 3D and 3F). At 3 dpi (n = 3), the average number of pyknotic nuclei was 4±1 cells/mm retina and 6±0 cells/mm retina in the ONL and INL, respectively. At 28 dpi (n = 5), the average number of pyknotic nuclei in the retinae was 6±1 cells/mm retina in the ONL and 4±1 cells/mm retina in the INL. On average, the RPE contained grade 4±1 vacuoles at 3 dpi and grade 2±0.5 vacuoles at 28 dpi (Fig 3D and 3F). With eye drop treatment at 3 dpi, the average grade of RPE vacuoles was reduced to 2±0.6.

### Delayed cell death following trauma

**Fig 4:**
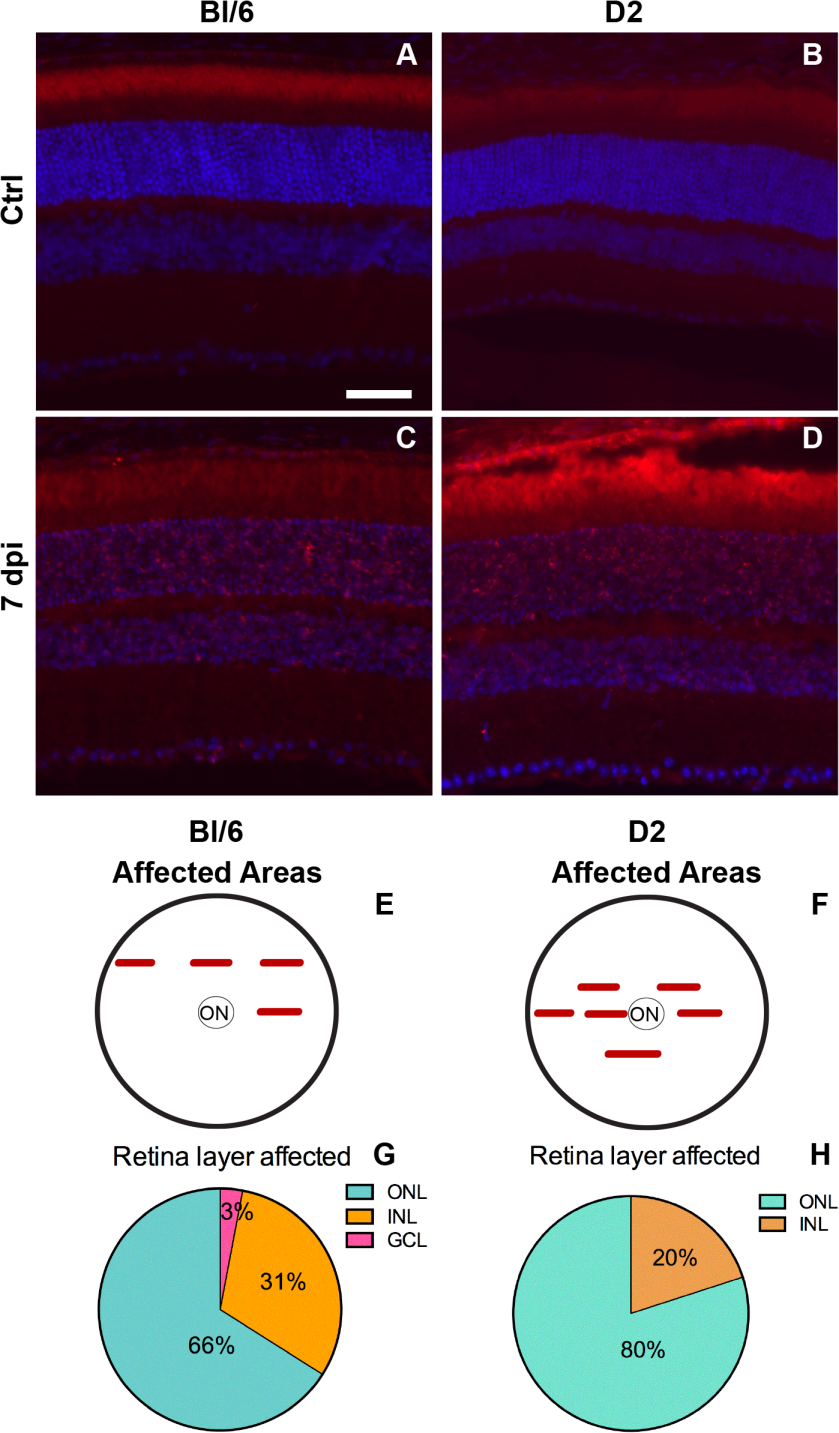
Cell death occurs in focal regions in both strains. Epifluorescence micrographs of retinal cross-sections from Bl/6 and D2 control eyes (A,B) and 7dpi Bl/6 and D2 eyes (C,D). The retina is labeled with TUNEL (red) and DAPI (blue). The scale bar in (E) is 50μm and applies to all micrographs in the figure. (E-F) Schematics illustrating the locations of retinal cross-sections containing TUNEL-positive cells, in relation to the optic nerve (ON) in the Bl/6 (E) and the D2 (F). (G-H) Pie charts showing the percentage of TUNEL-positive cells in each retinal layer in the Bl/6 (G) and D2 (H).

**Fig 5:**
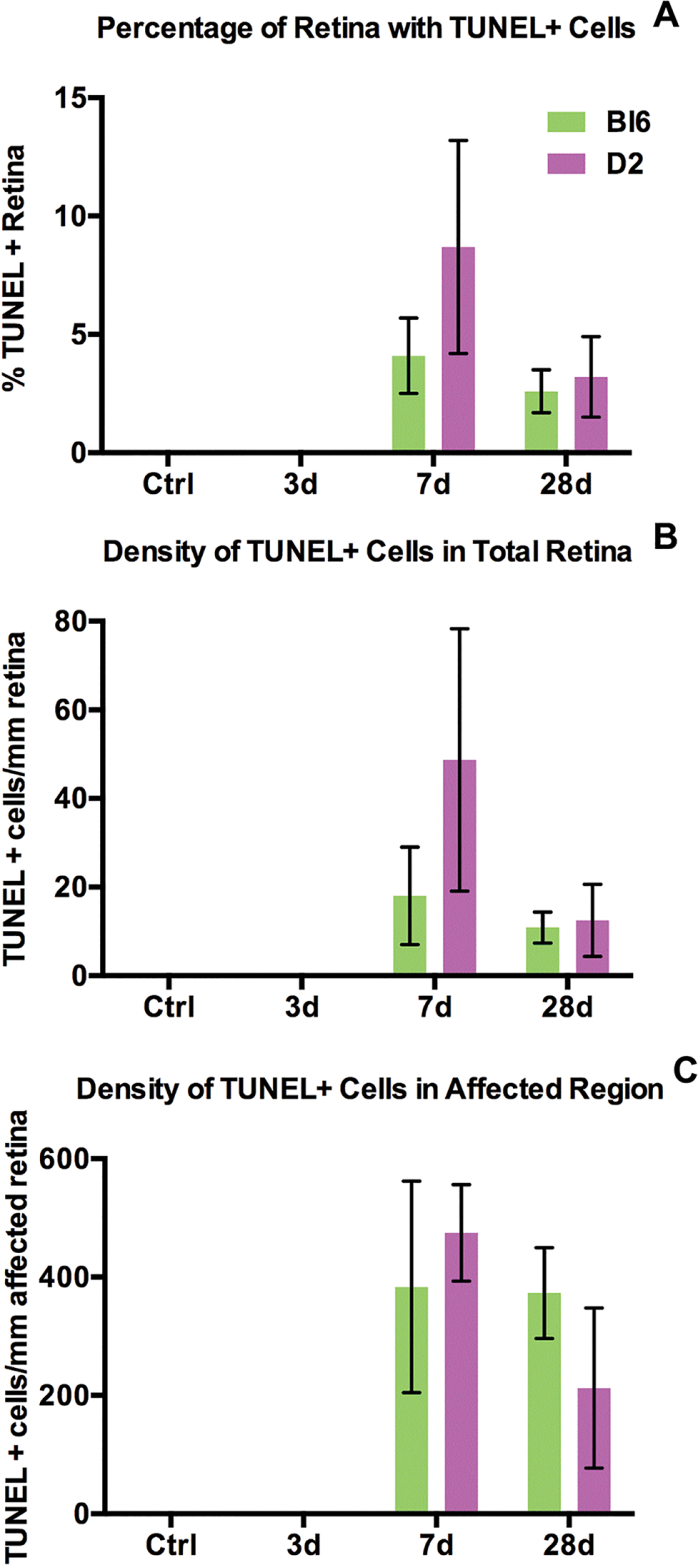
Cell death occurs at 7 and 28 dpi in both strains. Bar graphs show the percentage of retina containing TUNEL-positive cells (A), the density of TUNEL-positive cells in the retina (B) and the density of TUNEL-positive cells within the affected region (C). The bar graphs represent the average ± SEM for each time point and strain.

In the Bl/6 eyes post-injury, cell death was first detected at 7 dpi (n = 3) and was limited to small patches in the mid-peripheral and occasionally central retina (Fig 4C and 4E). The majority of the TUNEL-positive nuclei were located in the ONL (Fig 4G). TUNEL-positive cells were present in only 4±2% and 3±1% of the retina, at 7 and 28 dpi (n = 9), respectively (Fig 5A). The density of cell death throughout the entire retina was also low, with 18±11 cells/mm retina and 11±4 cells/mm retina, at 7 and 28 dpi, respectively (Fig 5B). Within areas of cell death (affected regions, Figs 4E and 5A), the density of TUNEL-positive nuclei was 383±179 cells/mm retina at 7 dpi and 373±77 cells/mm retina at 28 dpi (Fig 5C). The comparable density and percent retina affected at both time points suggests ongoing cell death in the Bl/6 from 7 to 28 dpi.

The onset of cell death in the D2 eye was also 7 dpi (n = 3) and was limited to small patches in the mid-peripheral and central retina (Fig 4D and 4F). Like the Bl/6, the majority of the TUNEL-positive nuclei were present in the ONL (Fig 4H). At 7 dpi, 19±8% of the retina contained TUNEL-positive cells (Fig 5A). At 28 dpi (n = 6), this decreased to 3±2% (Fig 5A). The density of TUNEL-positive nuclei throughout the entire retina of the D2 was low; 49±30 cells/mm retina at 7 dpi, decreasing to 12±8 cells/mm retina at 28 dpi (Fig 5B). The density of TUNEL-positive nuclei within the affected regions of the D2 retina was 475±81 cells/mm retina at 7 dpi and 212±135 cells/mm retina at 28 dpi (Fig 5C). The decrease in both the percent retina affected and the density of cell death in those areas at 28 dpi suggests that there is a peak of cell death at 7 dpi that decreases at 28 dpi.

### Necroptotic and pyroptotic pathways are activated following injury

**Fig 6:**
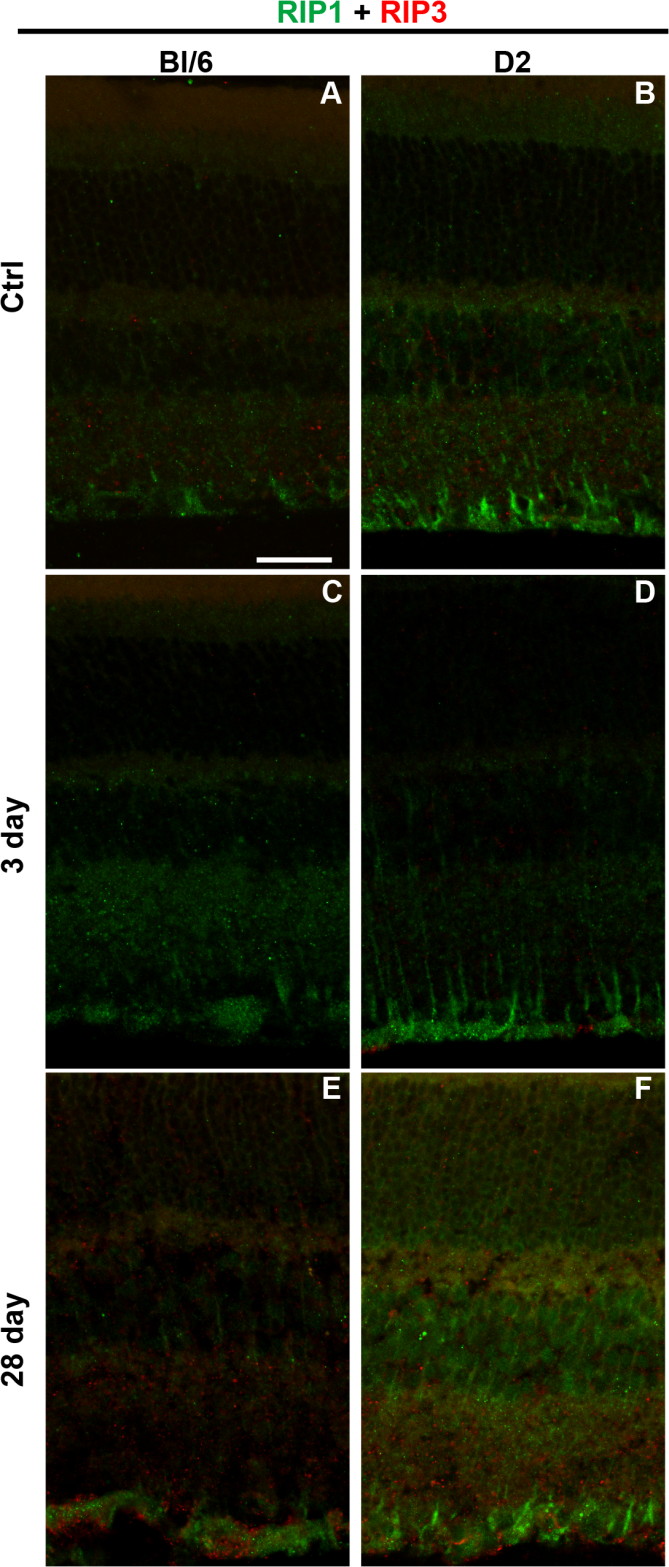
RIP1 and RIP3 immunolabeling increases after injury in both the Bl/6 and D2. Confocal micrographs of retinas from Bl/6 and D2 control eyes (A,B), Bl/6 and D2 3 dpi eyes (C,D) and Bl/6 and D2 28 dpi eyes (E,F). The scale bar in (A) is 50μm and applies to all micrographs.

In both Bl/6 and D2 control retinas, RIP1 localized primarily to the Müller glia, the INL, and the inner plexiform layer (IPL), with some light staining in the outer plexiform layer (OPL) (Fig 6A and 6B). RIP3 immunolabeling was present in the IPL and INL in control retinas from both strains (Fig 6A and 6B). At all time points post-injury, changes in RIP1 and RIP3 immunolabeling remained focal (e.g. limited to 3–4 sections per eye).

At 3 dpi (n = 5) in the Bl/6 retinae, RIP1 staining increased within the Müller glia and IPL, but there were no changes in RIP3 labeling when compared with controls (n = 3) (Fig 6C). In contrast, at 28 dpi (n = 4), RIP1 labeling was similar to baseline, but RIP3 labeling appeared in the ONL, IPL and GCL (Fig 6E). In the 3dpi D2 retina (n = 5) both RIP1 and RIP3 labeling was similar to controls (n = 3) (Fig 6D). At 28 dpi (n = 5), RIP1 labeling appeared brighter in the ONL and INL, while RIP3 labeling appeared brighter in all retinal layers (Fig 6F).

**Fig 7:**
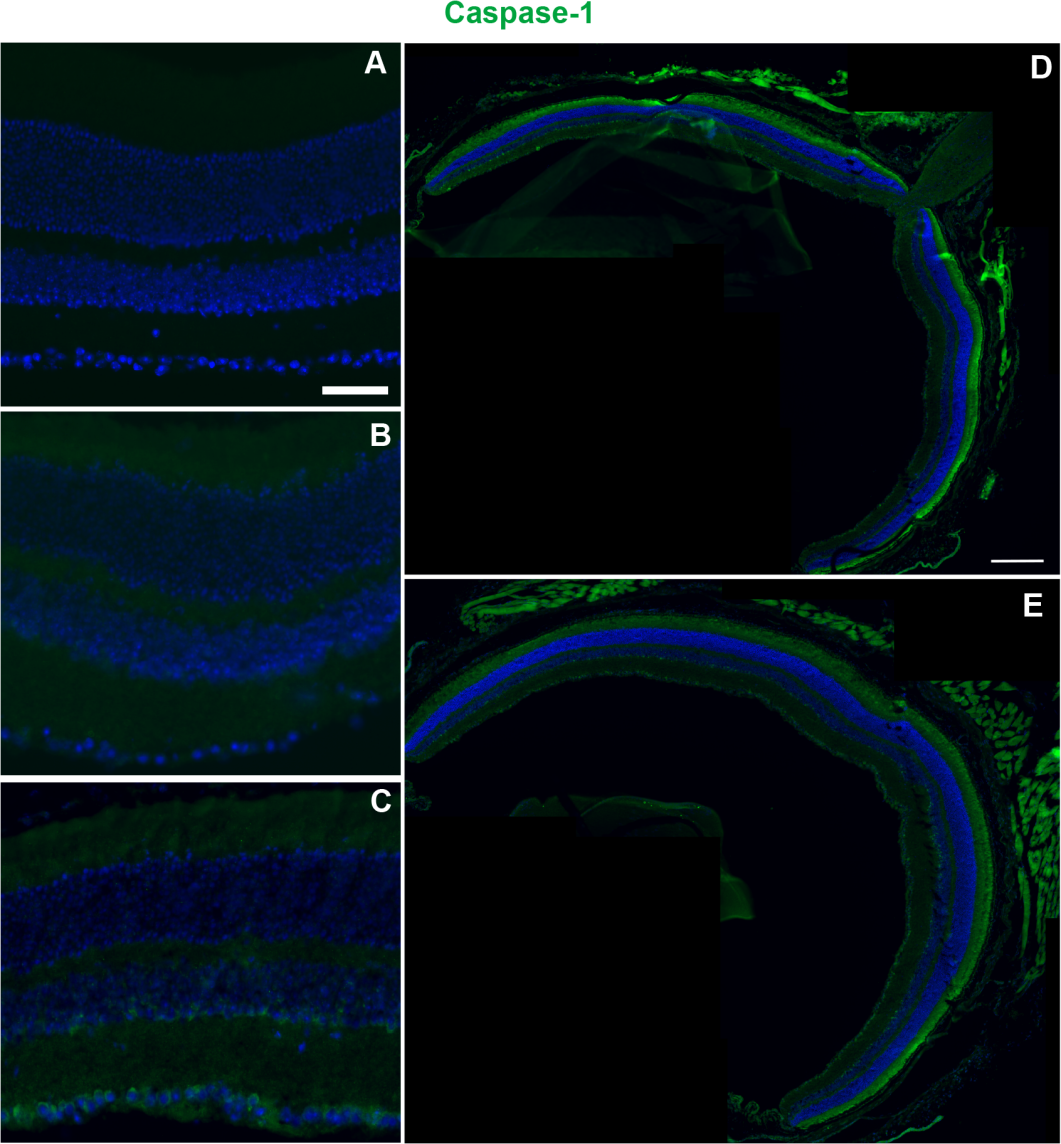
Caspase-1 immunolabeling increases at 28 dpi in the Bl/6 retina. Epifluorescence micrographs of retinas from control (A), 3 dpi (B) and 28 dpi (C) eyes labeled with anti-caspase-1 (green) and DAPI (blue). Caspase-1 immunolabeling at 28dpi is the same in both central (D) and mid-peripheral (E) retina. The scale bar is 50μm in (A-C) and 250μm in (D) and (E).

Caspase-1 immunolabeling was absent in control (n = 3) and 3 dpi Bl/6 retinas (n = 3) (Fig 7A and 7B). In contrast, the majority of 28 dpi Bl/6 retinas (n = 4) exhibited caspase-1 labeling in sparse cells at the inner edge of the INL and in the GCL (Fig 7C). The caspase-1 positive cells were present throughout the retina (Fig 7D and 7E). In the D2, caspase-1 positive cells were present within the INL and GCL of controls (n = 3) (**S1A Fig**). However, the caspase-1 labeling decreased after trauma such that no labeling was present at either 3 (n = 3) or 28 dpi (n = 3) (**S1B–S1C Fig**).

### Glial reactivity occurs after blast

**Fig 8:**
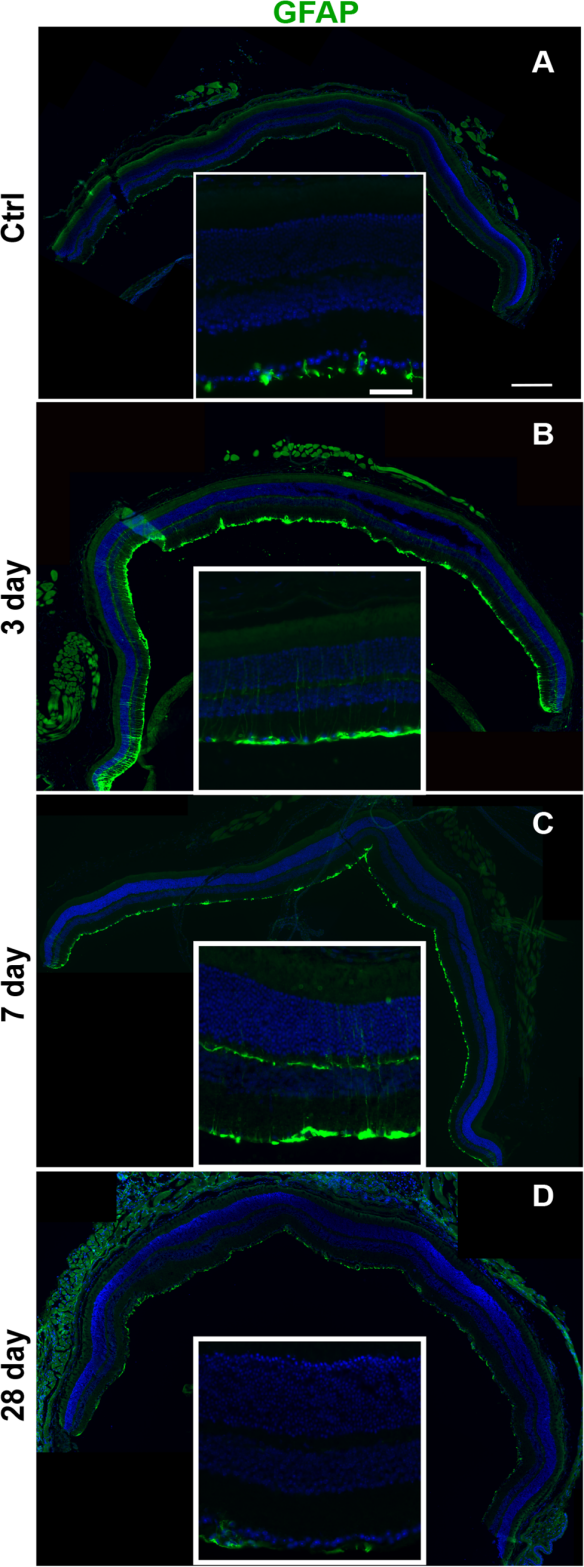
GFAP labeling is increased in the Müller glia of D2 eyes at 3 and 7 dpi. Low and high magnification epifluourescence micrographs of (A) control, (B) 3 dpi, (C) 7 dpi, and (D) 28 dpi retinas labeled with GFAP (green) and DAPI (blue). The scale bar for the high magnification micrographs is 50μm. The scale bar for the low magnification micrographs is 250μm.

As in the blast-exposed Bl/6 retina, Müller glia remained non-reactive at all time points (control n = 5, 3 dpi n = 6, 7 dpi n = 3 and 28 dpi n = 5), e.g. GFAP immunolabeling was restricted to the astrocytes and Müller glia endfeet (**S2 Fig**). In contrast, GFAP immunolabeling extended up the Müller cell processes in the D2 retinas (Fig 8B and 8C). At 3 dpi (n = 3), the GFAP positive processes were detected uniformly across the retina (Fig 8B). Treatment with non-medicated eye drops immediately post-blast reduced GFAP labeling to small patches in the mid-peripheral and central retina at 3 dpi (n = 4) (**S3B Fig**). At 7 dpi (n = 3), GFAP positive processes were only detected in small patches in the mid-peripheral and central retina (Fig 8C). Increased GFAP immunolabeling was not detected in any region of the retina at 28 dpi (n = 6) (Fig 8D).

**Fig 9:**
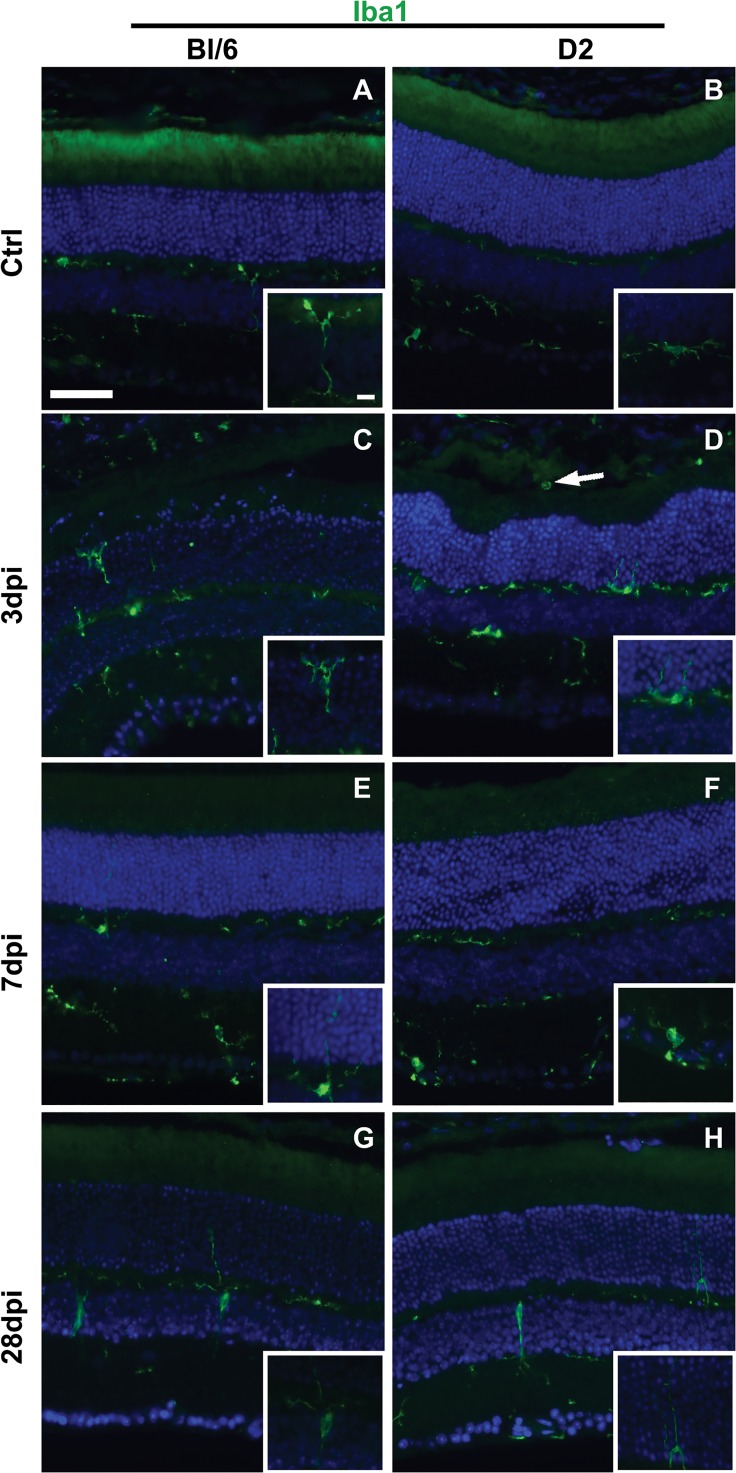
Reactive microglia are present in Bl/6 and D2 retinas after injury. Low magnification epifluorescence micrographs and high magnification micrographs (insets) of control (A-B), 3 dpi (C-D), 7 dpi (E-F) and 28 dpi (G-H) retinas immunolabeled with Iba1 (green) and DAPI (blue). The scale bar for the low magnification micrographs is 50μm. The scale bar for the inserts is 10μm.

In the normal Bl/6 (n = 5) and D2 (n = 5) retina, microglia had small somas with dendritic ramifications and were present only in the inner retina (Fig 9A and 9B). In the Bl/6 retina, all changes in microglial morphology after injury were limited to focal areas (e.g. 1–2 sections from each eye). At 3 dpi (n = 3), reactive microglia (enlarged somas with fewer shorter processes) were observed in the ONL and INL and microglial processes extended into the ONL (Fig 9C). At 7 (n = 3) and 28 dpi (n = 5), there were no reactive microglial somas in the ONL, but a few reactive microglia were present in the INL and occasional processes were detected in the ONL (Fig 9E and 9G).

Amoeboid, reactive microglia were commonly observed throughout the D2 retina at 3 dpi (n = 6) (Fig 9D). Macrophages were also occasionally observed in the outer segment layer of the photoreceptors in areas of retinal detachment (Fig 9D). At 7 (n = 3) and 28 dpi (n = 6), microglial reactivity was limited to focal areas of the inner retina in the mid-periphery and occasionally the central retina (Fig 9F and 9H).

### Protein nitration increases in the retina after blast

**Fig 10:**
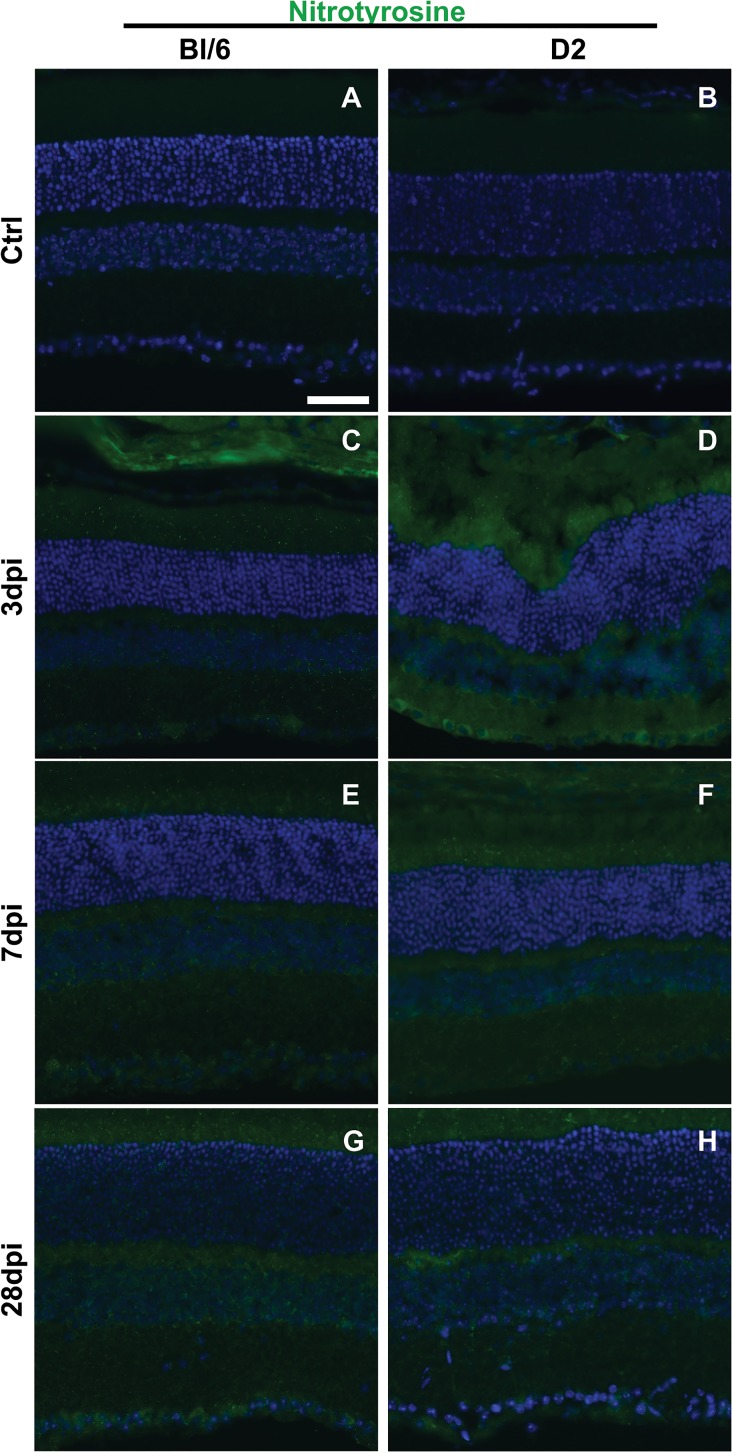
Nitrotyrosine immunolabeling increases after blast in the retina of both strains. Epifluorescence micrographs of control (A-B), 3 dpi (C-D), 7 dpi (E-F) and 28 dpi (G-H) retinal cross-sections labeled for nitrotyrosine (green) and DAPI (blue). The scale bar is 50μm and applies to all micrographs.

In the Bl/6 mouse, all eyes exhibited a slight increase in nitrotyrosine immunolabeling in the inner retina at 3 (n = 6) and 7 dpi (n = 3) when compared to controls (n = 3) (Fig 10C and 10E). Changes in nitrotyrosine immunolabeling were limited to focal areas within both the mid-peripheral and central retina. At 28 dpi (n = 5), there was nitrotyrosine immunolabeling in the outer retina (it is absent in the normal retina), in addition to more labeling in the inner retina (Fig 10G). The changes in labeling were uniform across the retina.

At 3 dpi (n = 7) in the D2 mouse, nitrotyrosine immunolabeling was greatly increased in the inner retina of all eyes with no apparent regional differences when compared to controls (n = 5) (Fig 10D). Labeling remained elevated throughout the inner retina of all eyes at 7 dpi (n = 3) and 28 dpi (n = 5), with no apparent regional differences (Fig 10F and 10H).

### Ocular trauma causes visual deficits

**Fig 11:**
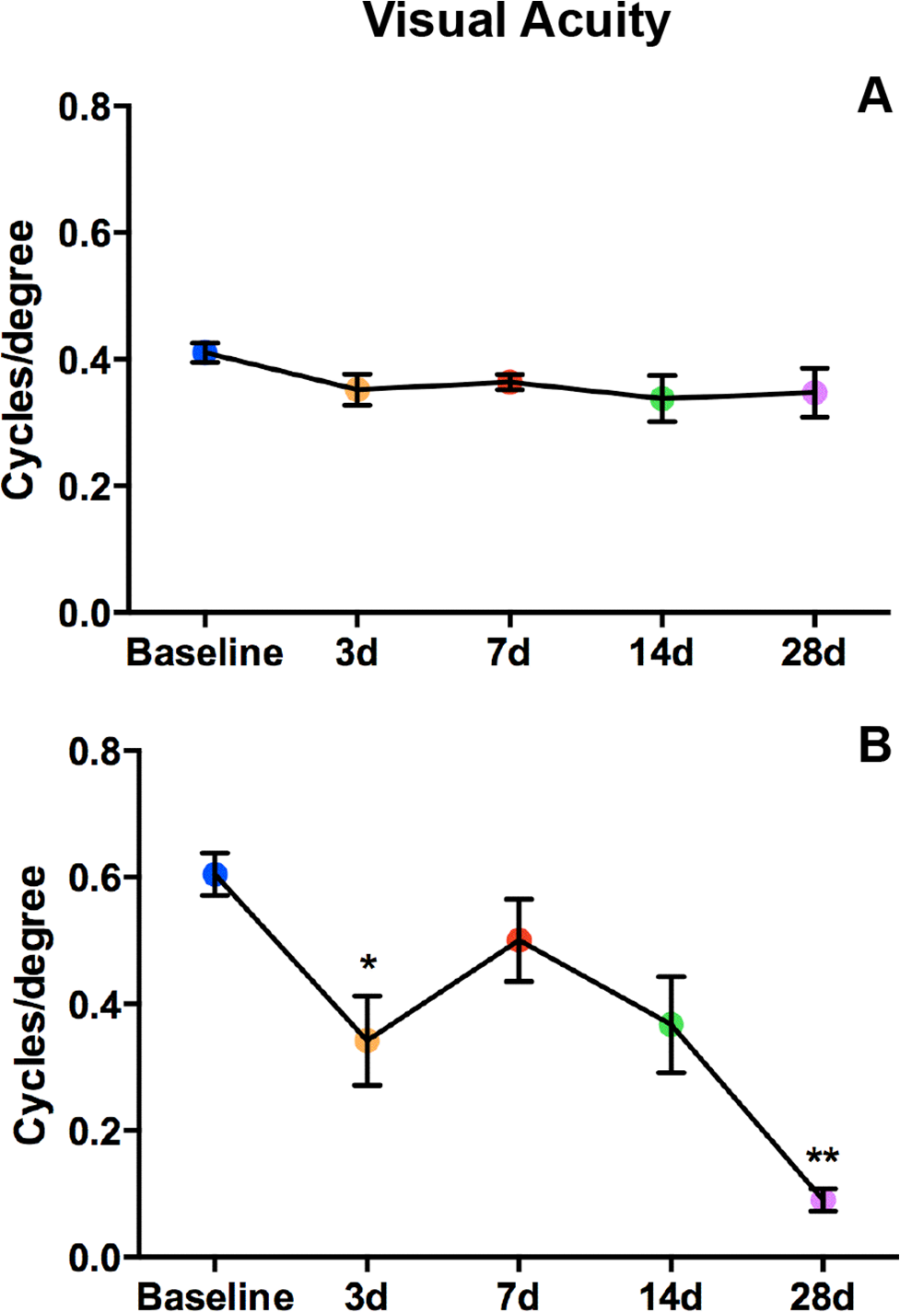
Visual acuity declines after blast in both strains. Graphs of OKN responses in Bl/6 (A) and D2 (B). The average cycles/degree ± SEM are plotted over time. *p<0.05, **p<0.01.

Both strains showed decreased OKN scores (i.e. visual acuity) in the contralateral eye post-blast (Fig 11A and 11B). There was no significant improvement in OKN scores over time, which normally occurs with retesting in this paradigm.[16] In the Bl/6 mouse (n = 16), OKN scores weren’t significantly different from baseline at any time point (Fig 11A). In the D2 mice (n = 9), OKN scores first declined significantly at 3 dpi (0.34±0.07 c/d, p<0.05) when compared to baseline values (0.60±0.03 c/d, Fig 11B). OKN scores were not significantly different from baseline at 7 and 14 dpi (0.50±0.06 and 0.37±0.07 c/d, respectively), but declined significantly at 28 dpi (0.09±0.02 c/d, p<0.0001).

**Fig 12:**
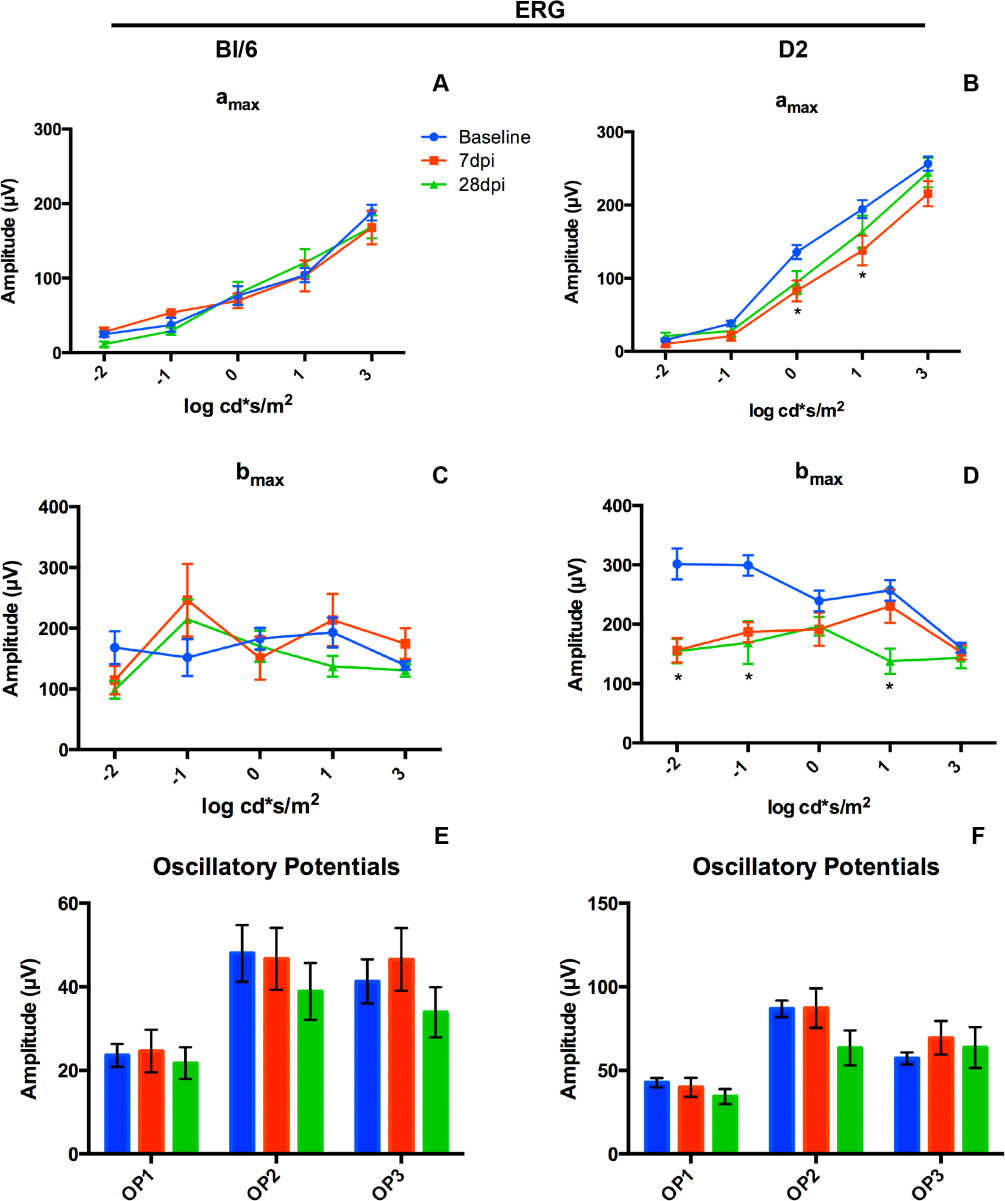
Blast causes ERG deficits in the D2, but not the Bl/6. (A,C,E) Graphs of the average ± SEM of a_max_ (A), b_max_ (C) and oscillatory potentials (E) for the Bl/6 mice at baseline, 7dpi and 28 dpi. (B, D, F) Graphs of the average ± SEM of a_max_ (B), b_max_ (D) and oscillatory potentials (F) for the D2 mice at baseline, 7dpi and 28 dpi. *p<0.05.

There were no significant changes in the ERG a_max_, b_max_ or oscillatory potentials at any time point assessed in the Bl/6 eye (n = 22) after injury (Fig 12A, 12C and 12E). However, there were significant reductions in both a_max_ and b_max_ in the D2 (n = 26) after blast when compared to baseline values (Fig 12B and 12D). At 7 dpi, the a_max_ was significantly lower than baseline at light intensities that correlate with daytime light levels. At 0 log cd*s/m^2^ the a_max_ was decreased by 39% from 135.7 ± 9.6 μV at baseline to 82.5 ± 14.2 μV, p<0.01 (Fig 12B). At 1 log cd*s/m^2^ the a_max_ was decreased by 29% from 194.3 ± 12.2 μV to 137.9 ± 20.2 μV, p<0.05 (Fig 12B). At 28 dpi, the a_max_ recovered, but the b_max_ was significantly reduced at several light intensities. At -1 log cd*s/m^2^ the b_max_ was decreased 44% from 299.0 ± 17.1 μV to 168.9 ± 35.8 μV, p<0.01 (Fig 12E). At 1 log cd*s/m^2^ the b_max_ was decreased 46% from 257.0 ± 17.1 μV at baseline to 137.7 ± 21.1 μV, p<0.01 (Fig 12E). The oscillatory potentials did not change after blast in the D2 (Fig 12E).

### Ocular trauma did not displace the lens

A possible explanation for the deficit in the OKN reflex but not in the ERG of the Bl/6 eye is altered optics. In order to determine if trauma caused lens displacement, we imaged and measured the depth of the anterior chamber in a subset of Bl/6 mice (n = 7) at baseline, 3–4 hours post-blast and 28 days post-blast, when the most profound deficits in the spatial acuity threshold were detected. There was no statistically significant difference in the anterior chamber depth between baseline (0.40±0.003 mm), 3–4 hours post-injury (0.39±0.005 mm) or 28dpi (0.40±0.006 mm).

### Ocular trauma did not damage the olfactory epithelium or optic nerves

The olfactory epithelium was examined in 7 dpi Bl/6 mice (n = 4) for evidence of blast injury to the tissue between the eyes (**S4 Fig**). We observed no differences between sham and blast animals with either nitrotyrosine immunolabeling (**S4A and S4C Fig**) or TUNEL (**S4B and S4D Fig**).

Since degenerative axons in the blast-exposed optic nerve were most evident at 28dpi, we examined contralateral optic nerves from both strains at 28 dpi (**S5 Fig**). There were no differences between them (**S5C and S5D Fig**) and control optic nerves (**S5A and S5B Fig**).

## Discussion

The mechanism of injury to the contralateral eye in this model system appears to be mild blunt trauma based on three main findings. First, absence of cell death and nitrosative stress in the olfactory epithelium argues against blast wave propagation through the head, as this would likely damage the delicate tissue. Second, the lack of damage to the contralateral optic nerve also supports this hypothesis since blast waves contain shearing forces that are particularly damaging to long structures such as axons [17]. Third, the timing of cell death was the same in the contralateral eyes of both mouse strains, but was different from the respective blast-exposed eyes [8,9]. One would expect similar timing of cell death if both eyes were injured by the same injury mechanism (i.e. blast).

Our injury system produced pathology similar to that observed in animal models of blunt ocular trauma and in patients with commotio retinae [18–21]. Commotio retinae is characterized by focal damage to the RPE and photoreceptors that spontaneously resolves in the majority of patients [18–21]. Similar to our findings, focal pyknotic photoreceptor nuclei were also detected soon after ocular trauma in both primate and rodent models [19,21]. Finally, porcine and feline models of blunt trauma also exhibit RPE vacuolization and focal photoreceptor damage within the first week post-injury, which resolved at one month post-injury [18,20]. Also consistent with trauma, we detected retinal detachments in both strains. Surprisingly, in the D2 mouse, we detected retinal detachments at 14, but not 7 days post-trauma. One possible explanation is that we missed the retinal detachments at 7 days due to how the mouse was positioned in the OCT system, or which retinal regions were imaged. However, since we rotate the mouse and probe to visualize as much of the retina as possible, we find this explanation unlikely. Another possible explanation is that retinal detachments can develop weeks after the initial injury. In fact, a recent study detected delayed retinal detachments in trauma patients [22].

In contrast with these other models, we also observed TUNEL-positive nuclei and labeling with necroptotic cell death markers in the inner retina. In a rat model of ocular trauma, the authors stated that the inner retina was mostly spared and did not report TUNEL quantification for the inner retina [21]. The pattern of their ERG deficits is also incongruous with our findings. They reported a deficit in both the *a*_*max*_ and *b*_*max*_ with equal timing and extent, while we saw an initial decrease in the *a*_*max*_, followed by a recovery of the *a*_*max*_ and subsequent decline in the *b*_*max*_ in the D2 mice. These findings suggest that the ERG deficits observed in their model were due to photoreceptor cell loss that resulted in loss of downstream signaling to the inner retina. In contrast, our ERG results show an early, transient deficit in the photoreceptor cells followed by inner retina dysfunction. The reasons for these differences are unclear.

The lower spatial acuity threshold scores in the Bl/6 eyes were consistent with our previous findings in the blast-exposed eyes [8]. While OKN scores normally improve with repeat testing in mice, there was no improvement in the Bl/6 eyes after injury [16]. The ongoing cell death from 7 to 28 dpi in the Bl/6 eyes may contribute to the decreased OKN response. Surprisingly, unlike in the blast-exposed eyes, there was no correlative attenuation of the ERG. The lack of lens displacement suggests that the discrepancy is not due to altered optics. It is feasible that the blunt trauma injured other, non-retinal, neuronal pathways necessary for the OKN response [23].

In contrast, the OKN scores and ERG results were very similar between D2 blast-exposed and contralateral eyes [9]. Also similar between eyes is the protective effect of the non-medicated eye drops. Since the eye drops are not medicated, it suggests that prevention of corneal damage was due to protecting the cornea from exposure/abrasion, i.e. exposure keratopathy. This fits with the timing and type of gross pathology detected in the D2 injured eyes. Tear film disruption has been reported in blast-exposed patients with traumatic brain injury [24]. Others have reported that damage to the cornea can cause an injury response (i.e. glial reactivity) in the retina [25]. Thus this may explain, in part, why greater glial reactivity and oxidative stress was seen in the D2 retina than in the Bl6 retina in the absence of eye drops and why these simple eye drops limited the area of retinal damage.

In conclusion, our model, which directs a blast of air towards one eye, causes injury to the contralateral eye likely as a result of pushing the head onto the cushioning within the housing chamber. The lack of ERG deficits, optic nerve damage, anterior pathologies and the spontaneous recovery of the outer retina damage suggest that this is a model of mild blunt trauma. The injury profile in the contralateral retina of the D2 mouse is more severe than that of the Bl/6 mouse, which is consistent with the greater neuroinflammation in the D2. Thus, our model system can be used to explore mechanisms and functional outcomes of both blast and blunt ocular trauma (e.g. damage from both the primary and tertiary effects of blast) separately. Future studies may investigate the effects of combining both blast and blunt injuries in the same eye, as this is relevant for blast-injured U.S. military veterans who often experience a combination of both primary and tertiary blast trauma.

## Supporting Information

S1 Fig Caspase-1 immunolabeling is reduced after trauma in the D2. Representative epifluorescence micrographs of control (**A**), 3 dpi (**B**) and 28 dpi (**C**) retinas immunolabeled for caspase-1. The scale bar in **A** is 50μm and applies to all micrographs.
(EPS)

S2 Fig GFAP immunolabeling remains restricted to the astrocytes and Müller cell endfeet in the Bl/6 after injury. Representative epifluorescence micrographs of control (**A**), 3 dpi (**B**), 7 dpi (**C**) and 28 dpi (**D**) retinas immunolabeled for GFAP. The scale bar in **A** is 50μm and applies to all micrographs.
(EPS)

S3 Fig Treatment with non-medicated eye drops reduces GFAP immunolabeling in the D2. Representative epifluorescence micrographs of non-eye drop treated (**A**) and eye drop treated (**B**) retinas labeled for GFAP (green) and DAPI (blue). The scale bar in **A** is 250μm and applies to both micrographs.
(TIFF)

S4 Fig Blast has no effect on the Bl/6 olfactory epithelium. Representative epifluorescence micrographs of sham (**A-B**) and 7 dpi (**C-D**) olfactory epithelium immunolabeled for nitrotyrosine (**A,C,** green) and TUNEL (**B, D,** red). The scale bar in **A** is 50μm and applies to all images.
(TIFF)

S5 Fig The optic nerve is unaffected by trauma. Representative micrographs of control (**A,B**) and 28 dpi (**C,D**) optic nerve cross-sections from Bl/6 (**A,C**) and D2 (**B, C**) mice. The scale bar in **A** is 20μm and applies to all micrographs.
(EPS)
