# Correction: Neurodegeneration and Vision Loss after Mild Blunt Trauma in the C57Bl/6 and DBA/2J Mouse

**DOI:** 10.1371/journal.pone.0165872

**Published:** 2016-10-27

**Authors:** Courtney Bricker-Anthony, Tonia S. Rex

Fig 11 is incorrect. The authors have provided a corrected version here.

**Fig 11:**
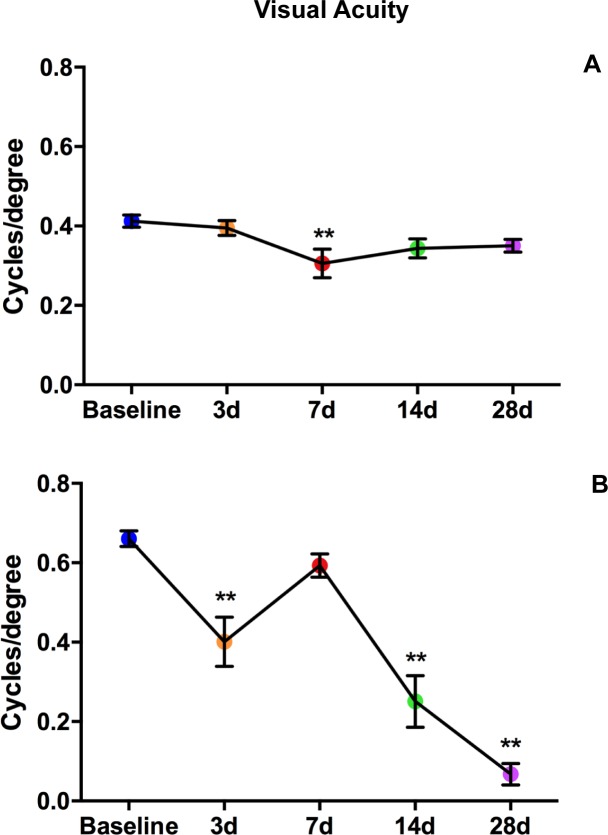
Visual acuity declines after blast in both strains. Graphs of OKN responses in Bl/6 (A) and D2 (B). The average cycles/degree ± SEM are plotted over time. *p<0.05, **p<0.01.

## References

[1] Bricker-Anthony C, Rex TS (2015) Neurodegeneration and Vision Loss after Mild Blunt Trauma in the C57Bl/6 and DBA/2J Mouse. PLoS ONE 10(7): e0131921 doi:10.1371/journal.pone.0131921 26148200 10.1371/journal.pone.0131921 PMC4493046

