# Supplementary figures and images for: Neurodegeneration and Vision Loss after Mild Blunt Trauma in the C57Bl/6 and DBA/2J Mouse
Source: PLoS One. 2015 Jul 6;10(7):e0131921. doi: 10.1371/journal.pone.0131921 (PMC4493046; doi:10.1371/journal.pone.0131921)

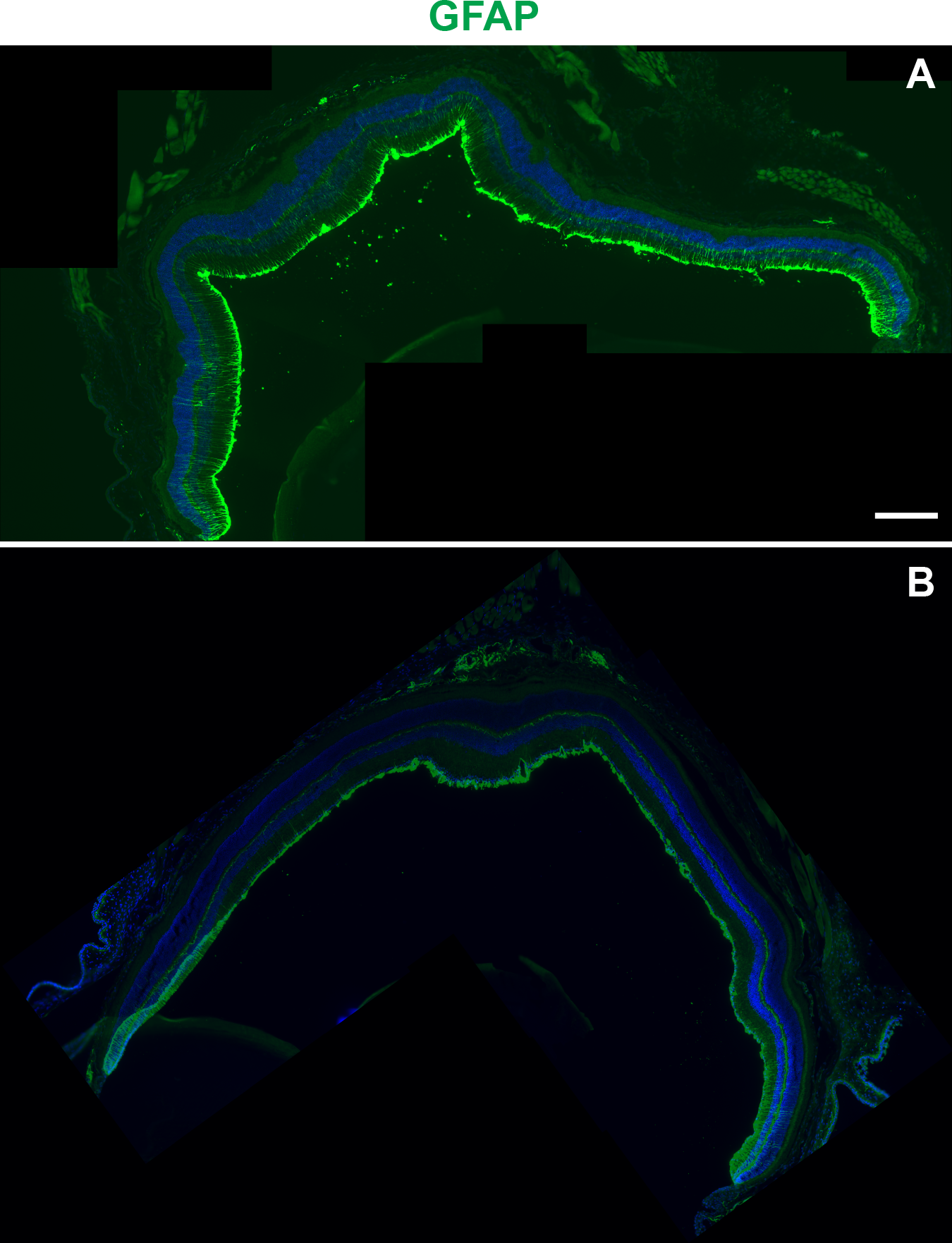

Supplement: S3 Fig — Representative epifluorescence micrographs of non-eye drop treated (A) and eye drop treated (B) retinas labeled for GFAP (green) and DAPI (blue). The scale bar in A is 250μm and applies to both micrographs. (TIFF) [file pone.0131921.s003.tiff]

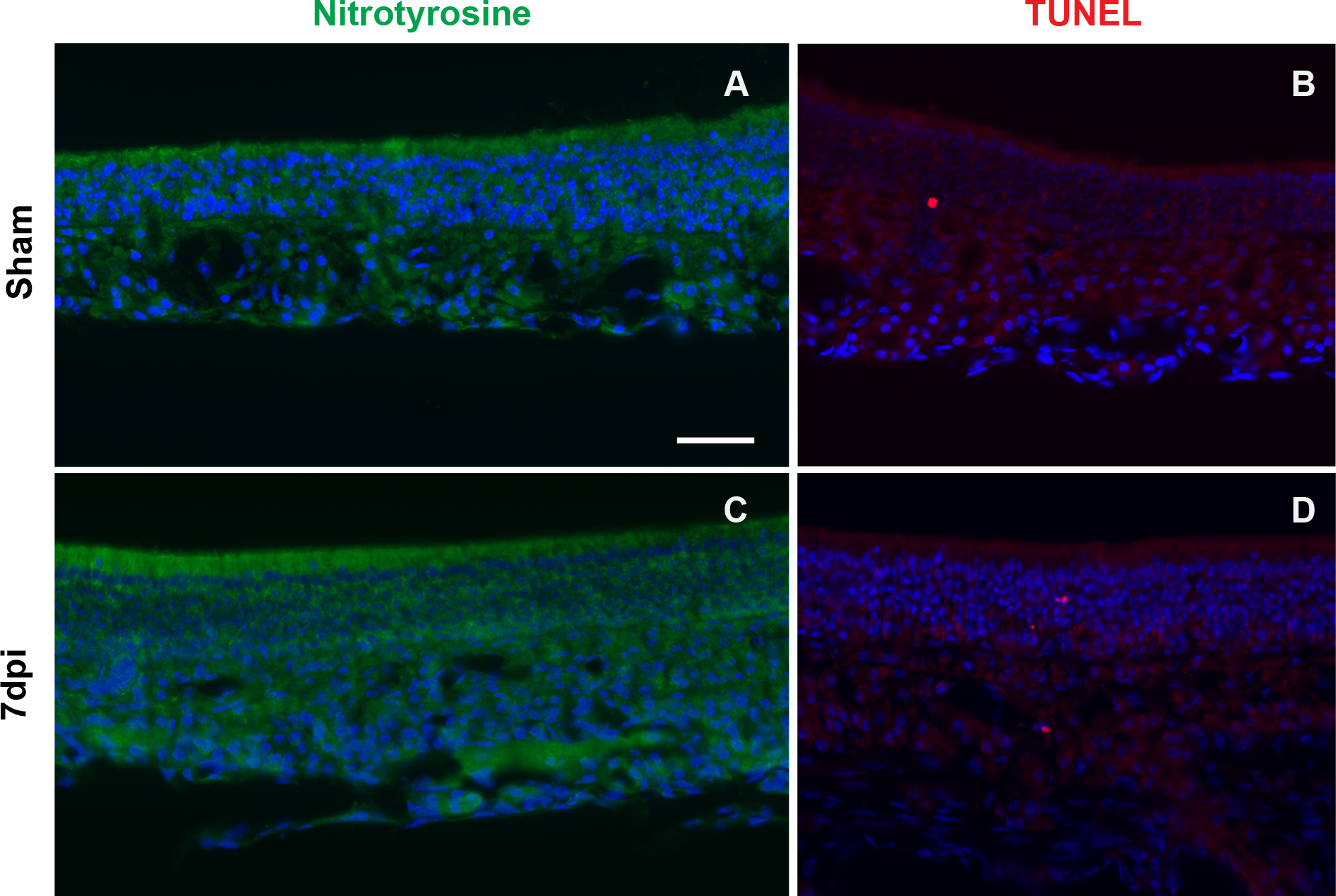

Supplement: S4 Fig — Representative epifluorescence micrographs of sham (A-B) and 7 dpi (C-D) olfactory epithelium immunolabeled for nitrotyrosine (A,C, green) and TUNEL (B, D, red). The scale bar in A is 50μm and applies to all images. (TIFF) [file pone.0131921.s004.tiff]
